# White Matter Neurons in Young Adult and Aged Rhesus Monkey

**DOI:** 10.3389/fnana.2016.00015

**Published:** 2016-02-22

**Authors:** Farzad Mortazavi, Xiyue Wang, Douglas L. Rosene, Kathleen S. Rockland

**Affiliations:** Department of Anatomy and Neurobiology, Boston University School of MedicineBoston, MA, USA

**Keywords:** interstitial, morphometrics, non-human primate, aging, Neu-N

## Abstract

In humans and non-human primates (NHP), white matter neurons (WMNs) persist beyond early development. Their functional importance is largely unknown, but they have both corticothalamic and corticocortical connectivity and at least one subpopulation has been implicated in vascular regulation and sleep. Several other studies have reported that the density of WMNs in humans is altered in neuropathological or psychiatric conditions. The present investigation evaluates and compares the density of superficial and deep WMNs in frontal (FR), temporal (TE), and parietal (Par) association regions of four young adult and four aged male rhesus monkeys. A major aim was to determine whether there was age-related neuronal loss, as might be expected given the substantial age-related changes known to occur in the surrounding white matter environment. Neurons were visualized by immunocytochemistry for Neu-N in coronal tissue sections (30 μm thickness), and neuronal density was assessed by systematic random sampling. Per 0.16 mm^2^ sampling box, this yielded about 40 neurons in the superficial WM and 10 in the deep WM. Consistent with multiple studies of cell density in the cortical gray matter of normal brains, neither the superficial nor deep WM populations showed statistically significant age-related neuronal loss, although we observed a moderate decrease with age for the deep WMNs in the frontal region. Morphometric analyses, in contrast, showed significant age effects in soma size and circularity. In specific, superficial WMNs were larger in FR and Par WM regions of the young monkeys; but in the TE, these were larger in the older monkeys. An age effect was also observed for soma circularity: superficial WMNs were more circular in FR and Par of the older monkeys. This second, morphometric result raises the question of whether other age-related morphological, connectivity, or molecular changes occur in the WMNs. These could have multiple impacts, given the wide range of putative WMN functions and their involvement in both corticothalamic and corticocortical circuitry.

## Introduction

The population of white matter neurons (WMNs; aka interstitial neurons) is considered to have an essential role in early development (Kanold and Luhmann, 2010; Wang et al., 2010). WMNs persist beyond early development but at a decreased density postnatally (Kostović and Rakic, 1980, 1990). In humans, there are multiple reports that psychiatric disorders such as schizophrenia are associated with altered density and distribution of WMNs (Kostović et al., 2011; Joshi et al., 2012; reviewed in Connor et al., 2011). Neuropathological changes in this population of neurons, as reported in multiple sclerosis (Chang et al., 2008), Alzheimer’s disease (McFadden and Minshew, 2013) and epilepsy (Loup et al., 2009; Liu et al., 2014), reinforce the possibility that these neurons may be selectively susceptible to oxidative stress or other toxicity.

In addition to their likely importance in neuropathological conditions, key features of this rather neglected population of neurons are: (1) they are phylogenetically conserved, and have been identified in adult rodents, carnivores, primates, and cetaceans, although with pronounced species differences (Reep, 2000; Clancy et al., 2009; Suárez-Solá et al., 2009; Kanold and Luhmann, 2010; Hoerder-Suabedissen and Molnár, 2015); (2) they are more abundant in larger, gyrencephalic brains, although at lower numbers in the adult than in the prenatal or neonatal brain; (3) they are neurochemically and morphologically heterogeneous, within and across species (Smiley et al., 2000; Suárez-Solá et al., 2009; García-Marin et al., 2010; Judaš et al., 2010; Raghanti et al., 2013; Hoerder-Suabedissen and Molnár, 2015); (4) they are incorporated into the circuitry of the overlying cortex, as shown by retrograde tracing experiments (Tomioka et al., 2005; Tomioka and Rockland, 2007) and by intracellular fills *in vitro* (Clancy et al., 2001; von Engelhardt et al., 2011); and (5) an association has been established with vasodilation (Estrada and DeFelipe, 1998; Cauli and Hamel, 2010) and sleep regulation (Kilduff et al., 2011). Given that the population consists of different cell types (in monkeys: Gabbott and Bacon, 1996a,b; Delalle et al., 1997; in humans: Delalle et al., 1997; Suárez-Solá et al., 2009; García-Marin et al., 2010), the functional roles are likely to be multiple and diverse.

Given the potential importance of WMNs and the documented changes in their density under pathological conditions, the current investigation set out to investigate whether there is an age-related decline in density of WMNs in the rhesus monkey model of normal aging. In specific, we were motivated by the relative lack of data for this population in non-human primates (NHP) and the idea that changes in this population might offer a specific assay for aging and pathological conditions.

Stereological investigations in monkeys have consistently reported that there is no age-related cell loss in cortical gray matter (e.g., Hof et al., 2000; Giannaris and Rosene, 2012), while substantial age-related changes in myelinated axons are well established (Kohama et al., 2012). As neurons, WMNs might follow with neurons in the gray matter and show no change in number. Alternately, their close association with the white matter environment, vulnerable to inflammation and oxidative stress in normal aging, might influence age-related changes including cell loss. Along these lines, there is a significant age-related numerical increase reported for glia in the infragranular layers in visual cortex. This was suggested to reflect a glia response to pathology in the surrounding myelinated projection fibers (Giannaris and Rosene, 2012).

It was thus hypothesized that degeneration of myelin that is observed in the aging monkey could potentially affect patterns and distributions of WMNs. Accordingly, regional density of superficial and deep WMNs in frontal (FC), temporal (TE), and parietal (Par) association cortices was compared in four young adult and four aged rhesus monkeys (Table 1). These association areas were chosen for the sake of more direct comparison with neuropathological changes in humans. The occipital lobe was not used because it has been reported as an outlier, with lower numbers of WMNs (Smiley et al., 1998; García-Marin et al., 2010). In addition several morphometric measures were used to further characterize and assess differences in superficial and deep WMNs in this cohort of animals.

**Table 1:** **Subject data**.

Subject	Sex	Age (years)
Am099	M	6
Am204	M	6.1
Am111	M	6.8
Am227	M	7.8
Am189	M	24.9
Am226	M	20.5
Am236	M	22.9
Am207	M	28.7

## Materials and Methods

### Subjects

This study utilized tissue from eight male rhesus monkeys (*Macaca mulatta*) that were part of previous studies on normal aging (Table 1). The monkeys were obtained from national primate research centers and all had known birth dates and complete medical records. Monkeys were housed at the Laboratory Animal Science Center at Boston University Medical Center (BUMC), which is fully accredited by the Association for the Assessment and Accreditation of Laboratory Animal Care (AAALAC). All procedures were performed according to protocols reviewed and approved by the BUMC Institutional Animal Care and Use Committee. All monkeys underwent a battery of behavioral tests to assess cognitive function (Herndon et al., 1997; Moss et al., 1997; Moore et al., 2006). Once testing was completed and prior to perfusion, all monkeys received magnetic resonance imaging (MRI) scans to ensure that there was no occult brain damage.

### Tissue Preparation

**Figure 1:**
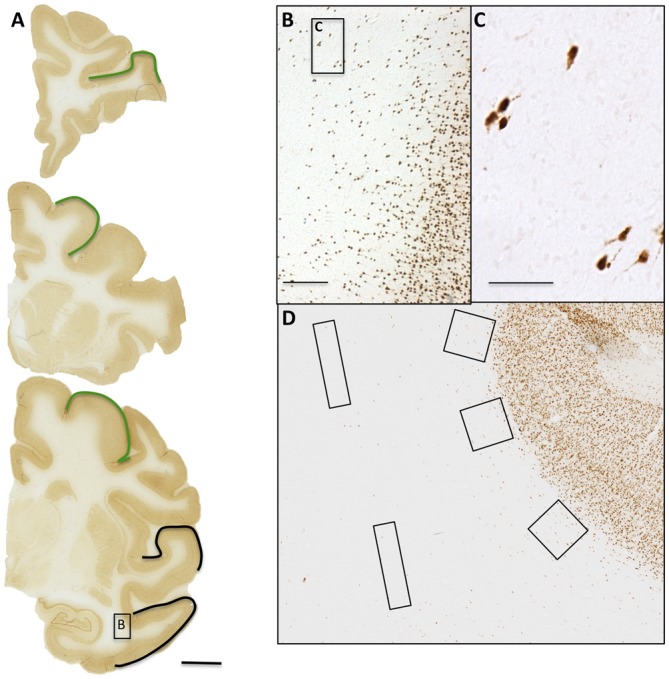
**WMNs are visualized by Neu-N. (A)** Three coronal sections from the frontal, parietal, and temporal areas. Regions of interest (ROIs) are identified by outline (top and middle: frontal; bottom: parietal in green and temporal in black). **(B)** Representative field of Neu-N immunolabeled neurons, from the boxed temporal region in the coronal section at bottom. **(C)** Higher magnification from the box in **(B)**. **(D)** Representative placement of three counting boxes (400 × 400 μm) along the superficial white matter (WM) and two rectangles (800 × 200 μm) within the deep WM. Scale bars = 500 μm in **(A)**; 250 μm in **(B)**; 50 μm in **(C)**.

Monkeys were tranquilized with ketamine (10 mg/kg, intramuscular), and deeply anesthetized with sodium pentobarbital (15 mg/kg, intravenous to effect), before euthanasia by exsanguination during transcardial perfusion of the brain. All monkeys were perfused with 4% Krebs buffer at 4°C, followed by 4% paraformaldehyde in phosphate buffer (0.1 M, pH 7.4) at 37°C. Brains were blocked *in situ*, in the coronal plane just behind the splenium, removed from the skull, weighed and post-fixed overnight in 4% paraformaldehyde for no more than 18 h, and transferred to cryoprotectant solution to eliminate freezing artifact (Rosene et al., 1986). Blocks were flash frozen at −75°C and stored at −80°C until they were cut on a microtome. The cutting protocol consisted of a repeated series of coronal sections, eight of which were 30 μm thick and one 60 μm thick, resulting in a spacing of 300 μm for sections within each series. Then, one half-series of sections from these animals was used for the current study. Within this half series, we used 3–4 sections from each animal for each field, FC, Par, and TE (Figure 1), corresponding to sections from Bregma: +8.10, +0.05, −5.85, and −10.35 mm (Paxinos et al., 2000).

Other tissue was available from a previous study (Rockland and Nayyar, 2012) in adult macaque monkeys, which had been histochemically reacted for NADPH- diaphorase. This served as a useful confirmation for soma size and shape.

### Immunohistochemistry

Sections from each of the eight subjects were thawed together and rinsed three times for 5 min in 0.05 M Tris-buffered saline (TBS) to wash off the cryoprotectant. All sections were then processed at the same time and in the same reagents to minimize procedural variability. To quench endogenous peroxidases, sections were incubated for 30 min in 0.05 M TBS containing 1% hydrogen peroxide. After three 5 min washes in 0.05 M TBS, sections were incubated for 1 h in a blocking solution of 10% Normal Goat Serum (NGS) and 0.4% Triton-X in 0.05 M TBS. The sections were incubated for 48 h at 4°C with gentle agitation in a mouse anti-Neu-N IgG (1:10,000; MAB377, Chemicon, Temecula, CA, USA) in 0.05 M TBS, containing 2% NGS, and 0.1% Triton-X. Following incubation with the primary antibody, the sections were washed three times for 5 min in 0.05 M TBS with 2% NGS and 0.1% Triton-X, followed by a 2 h incubation with the secondary antibody (goat anti-mouse, 1:600; Vector, Burlingame, CA, USA) in 0.05 M TBS containing 2% NGS, and 0.4% Triton-X. The sections were then washed three times for 5 min in buffer and subsequently incubated with an avidin biotinylated horseradish peroxidase complex for 1 h. The sections were washed again with three 5 min washes in 0.05 M TBS. All sections were incubated together for 7 min in sodium acetate containing 0.55 mM 3–3′-diaminobenzidine (DAB; Sigma, St. Louis, MO, USA) and 0.01% H_2_O_2_. The sections were washed three times for 5 min each in 0.05 M TBS and mounted onto gelatin-coated slides, air dried, and cover-slipped with Permount mounting medium (Thermo Fisher Scientific, Waltham, MA, USA).

### Estimation of WMN Density in FC, TE, and Par Association Cortices

Designed-based stereological analysis necessitates defining boundaries of a region of interest (ROI) for systematic random sampling from equally spaced sections throughout the structure of interest. Importantly, estimates of volume or cell population in a ROI are dependent on delineation of boundaries. This requirement, while often feasible for gray matter measurements, is less so for white matter since there is no map equivalent to Brodmann areas for white matter and delineating ROIs in relation to overlying gray matter would be at best approximate. Accordingly, we adopted a broad regional approach, similar to García-Marin et al. (2010), where WNN density was assessed according to frontal, parietal, or temporal regions (Figure 1).

**Figure 2:**
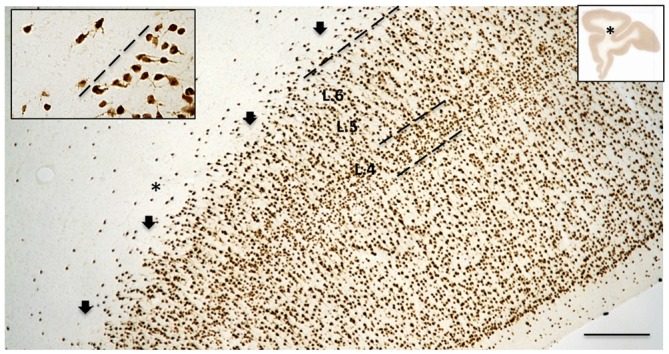
**The border between layer 6 (L. 6) and the superficial WM is typically broken or scalloped, with cell-sparse gaps (arrows), as seen in this coronal section from the upper bank of the principals sulcus (asterisk in the low magnification section, inset at right; from case 189).** Dashed line indicates border between L. 6 and the superficial WM. The borders of layer 4 (L. 4) are also indicated. Higher magnification inset at upper left illustrates lower density of neurons in the WM, as compared with L. 6, as well as their tendency for more horizontal orientation. Scale bar = 300 μm for the large-field and 75 μm for inset.

Another issue in counting WMNs is the need to distinguish the boundary between superficial white matter and layer 6, and between superficial and deep white matter (Figure 2). The boundary with layer 6 can be estimated by a fall-off in cell density, and a change in soma morphology, where there is a decreased proportion of pyramidal neurons. Care was taken to avoid zones where presumptive WMNs could be confused with tangentially sectioned layer 6. There are fewer guidelines for the designation of deep white matter, except for the continued decline in cell density and the greater distance from the overlying gray matter.

To estimate density and regional distribution of WMNs, Neu-N labeled sections were digitized and sampling boxes overlain (Figure 1), in a semi-systematic approach similar to that of Xu et al. (2011), Kinney et al. (2012), and Yang et al. (2011). In specific, 3–4 slides per region (FC, Par, and TE) were digitized as whole brain hemisphere photomontages, using the 10× objective on an E600 Nikon microscope equipped with Turboscan Montaging system (Objective Imaging, UK). Using ImageJ (version 1.47, NIH, Bethesda, MD, USA) software to process all images identically, ROIs for superficial and deep WM were superimposed with sampling boxes (400 × 400 μm in superficial; 800 × 200 μm in deep WM). This approach yielded 20–30 counting boxes per region (Figure 1) for each subject; that is, a total number of 1275 unbiased counting boxes. Density of neurons in superficial and deep WM was then calculated by counting the number of neurons per total area sampled. An experimenter blind to the age of the monkeys and identity of the ROIs performed the counting from the acquired images. The advantage of the Turboscan Montaging system is that the person performing the counts can zoom in at high-resolution to the overlaid boxes for counting the cells. As a control for accuracy, density measurement counts were replicated in 1 or 2 sections from a subset of three different animals. This procedure resulted in an Interclass Correlation Coefficient of 0.93.

In addition, for two brains, stereological methods were also applied, using a standard sampling grid (400 × 400 μm). This method was less suitable since: (1) a large subsample of boxes was not parallel to the pia surface, contained a portion of layer 6 neurons, and thus were discarded; and (2) superficial and deep white-matter were also “straddled” by large portions of the grid. When neurons from layer 6 were excluded from what was intended as “systematic random sampling, ” the number of superficial and deep WMNs was within 10% of the sampling method used for the whole cohort of animals.

### Morphometric Analysis of WMNs

High-resolution images acquired with Turboscan from four monkeys (two young adult and two aged) were used for the morphometric analysis. A distinct advantage of the Turboscan system is that each tile acquired is at its best depth of focus, thus producing images as similar as possible to a “maximum projection” (our sections, while cut at 30 μm, typically dry to about 12–18 μm in thickness after processing and dehydration). Table 2 shows the numbers of WMNs in each strata that were used for quantification of morphometrics. Using ImageJ, the high-resolution images from each ROI were first converted to an 8-bit image; and an experimenter blind to region and to age of the animal, thresholded the 8-bit image and then converted it to a binary image. The experimenter also had an original photomicrograph to monitor the threshold intensity based on single neuron profiles. Three measures were used in the morphometric analysis: Soma Area, Soma Perimeter, and Soma Circularity. Circularity [4π (area/perimeter^2^)] was a measure of how closely a given soma shape matches a perfect circle and ranges from 0, an infinitely elongated polygon, to 1, a perfect circle.

**Table 2:** **Numbers of neurons used for morphometric analysis**.

	Superficial		Deep	
	Young (*n* = 2)	Old (*n* = 2)	Young (*n* = 2)	Old (*n* = 2)
Frontal	428	469	63	68
Parietal	574	517	109	118
Temporal	700	716	129	134

### Statistical Analysis

For the analysis of density of WMNs, as a first step we tested for normality and homoscedasticity of the distribution of WMNs in each zone using Shapiro-Wilk test for normality. The Shapiro-Wilk test of normality for the distribution of data showed that the counts in each strata are not normally distributed (skewed distribution, Figure 2). Specifically, Krustal-Wallis test (non-parametric counterpart to dependent *t*-test) was used to determine differences in numbers of neurons in superficial vs. deep strata. To determine if age-related differences existed in these strata, a Mann-Whitney *U*-test (non-parametric counterpart to independent sample *t*-test) was used. Finally, a Friedmann’s test (non-parametric counterpart to ANOVA) was used to determine overall density differences in each ROI. This was followed up with Bonferroni correction for multiple comparisons.

The morphometric characteristics were also tested for normality. Since these met the necessary assumption of normality, parametric statistical tests were used. A 2 (young vs. old) × 2 (superficial vs. deep) × region (FC, Par, TE) multivariate ANOVA with Bonferroni correction for *post hoc* analysis was used. The data were analyzed using the Statistical Package for Social Sciences software (SPSS, version 19.0, Chicago, IL, USA). All results are reported as mean ± SEM, unless stated and alpha was set at 0.05.

## Results

### Visualization of WMNs

WMNs were visualized by immunohistochemistry (IHC) for Neu-N (Figures 1, 2). Importantly, Neu-N allows small neurons to be clearly distinguished from similar sized glia, a distinction that is often difficult in standard cellular Nissl stains. Three zones of WMNs were distinguished; namely: (1) putative WMNs immediately below but possibly intermingled with layer 6; (2) those more clearly in the superficial WM, defined as a 400 μm zone below determined layer 6; and (3) those in the deep WM, defined as more than 500 μm below layer 6. These boundaries were adopted as convenient, but are subject to further refinement. For example, the “deep” WM in a gyral context may be better viewed as the merging of two zones of superficial WM. In contrast with the relatively distinct subplate layer (“layer 7”) in rodents, the superficial WM in NHP is, typically broken or scalloped, with small cell-sparse gaps (about 150 μm across; Figure 2). In determining the border between layer 6 and the superficial WM, we took account of diminished cell density in the superficial WM and a trend toward more horizontally oriented somata for superficial WMNs (Figure 2).

### Quantification of WMNs by Strata and Age

**Figure 3:**
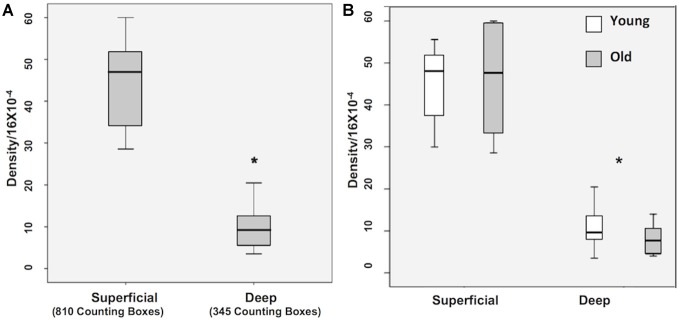
**Box plots of WMN density from Superficial and Deep zones in young (*n* = 4) and old (*n* = 4). (A)** Box plot showing the non-normal distribution of white matter neurons (WMNs) in both strata. **(B)** Significant differences in the number of neurons between the strata, but not between the young and old monkeys. *p < 0.05, compared to superficial.

A Krustal-Wallis test showed that there are significantly less WMNs in the deep zone (Median = 9.5), compared to the superficial zone (Median = 47.5; *Z* = −3.657, *p* < 0.05; Figure 3A). While a significant difference in the number of WMNs in each strata was observed, a Mann-Whitney U test (non-parametric counterpart to independent sample *t*-test) showed no significant age differences between the young (*n* = 4) and older monkeys (*n* = 4) in the overall counts in either strata (Figure 3B).

### Regional Density of WMNs in the Three ROIs

**Figure 4:**
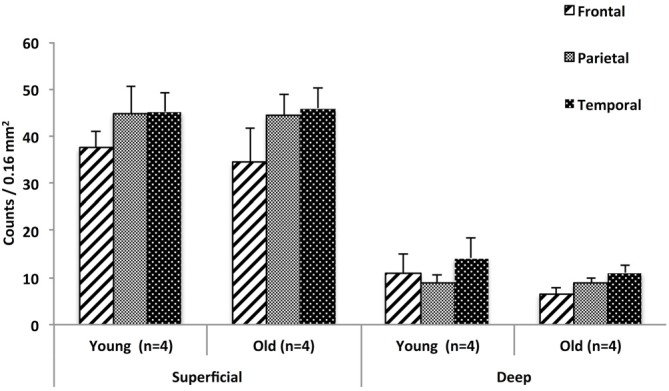
**Regional distribution of WMNs in frontal (FR), parietal (Par), and temporal (TE) in young (*n* = 4) and old monkeys (*n* = 4).** No significant difference was observed in the density of WMNs in the three regions, nor between the age groups.

As shown in Figure 4 the number of superficial Neu-N-immunoreactive (ir) WMNs per 0.16 mm^2^ was determined as about 40 in the superficial, and about 10 neurons for the deep WM ROIs. This was consistent for all three regions. For all three, there was no significant difference between young and old animals. Across the three regions, the density varied slightly (36–45) in the superficial and 8–12 in the deep (zone), with a trend for lower density in the frontal region of the older monkeys (Figure 4).

**Figure 5:**
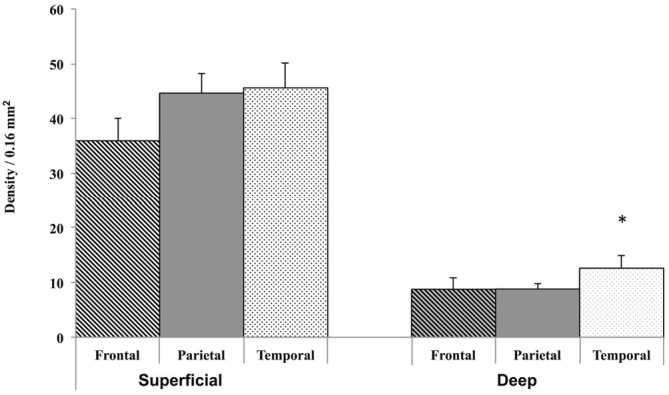
**Grouped data from young and old monkeys (*n* = 8) only showed a significant difference for the density of WMNs in the deep strata in TE, but not for those in FR and Par, nor for superficial WMNs in any of the other regions.** *p < 0.05, compared to all others.

Finally, since there were no significant age differences in the density of WMNs between the young and old monkeys, we pooled the density measurements of the young and old group for analyzing the regional distribution. A Friedmann’s test (non-parametric alternative to ANOVA) showed significant differences in the density of deep WMNs in the three different regions (FC, TE, and, Par) [***λ***^2^_(8)_ = 7, *p* < 0.05] but not in the superficial zone. *Post hoc* analysis with Bonferroni correction for multiple comparisons showed significantly more deep WMNs in TE (12.6 ± 2.30) compared to FC (8.8 ± 1.10) and Par (8.8 ± 1.02; Figure 5).

### Morphometrics of Superficial and Deep WMNs

While no significant age-related differences were observed in cell density, there were several morphometric differences that were statistically significant. A 2 (young vs. old) × 2 (superficial vs. deep) × 3 (FR, Par, and TE) multivariate ANOVA with Bonferroni adjustment for multiple comparisons showed a main effect of age, on three morphometric measurements. Soma area [*F*_(1,4012)_ = 5.77, *p* < 0.05] and perimeter [*F*_(1,4012)_ = 3.992, *p* < 0.05] were larger in young animals compared to the soma size from older monkeys. Soma shape was less circular [*F*_(1,4012)_ = 6.856, *p* < 0.05] in young vs. older monkeys.

The ANOVA also showed a main effect of strata, where the three morphometric measures were statistically different in superficial compared to the deep strata. Overall, somata in the superficial zone are larger in area [*F*_(1,4012)_ = 930.50, *p* < 0.05] and perimeter [*F*_(1,3276)_ = 450.211, *p* < 0.05] but less circular [*F*_(1,3276)_ = 19.67, *p* < 0.05]). A main effect of ROI was also observed; namely, regional analysis of soma morphology showed differences in soma area [*F*_(2,4012)_ = 15.18, *p* < 0.05], perimeter [*F*_(2,4012)_ = 3.96, *p* < 0.05] and circularity [*F*_(2,4012)_ = 6.82, *p* < 0.05] across the three regions (FR, Par, TE).

**Figure 6:**
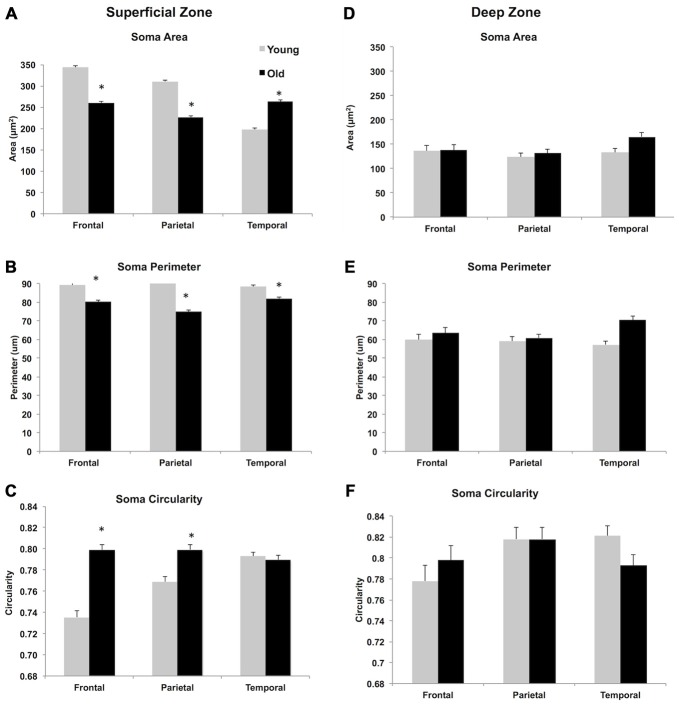
**Morphometric analysis of WMN in superficial (A–C) and deep zones (D–F).** The widest range in area and perimeter and shape was observed in superficial WMNs of the temporal lobe. In the deep white matter, neurons showed greater variability in size and shape in all three regions. *p < 0.05, compared to young monkeys.

**Figure 7:**
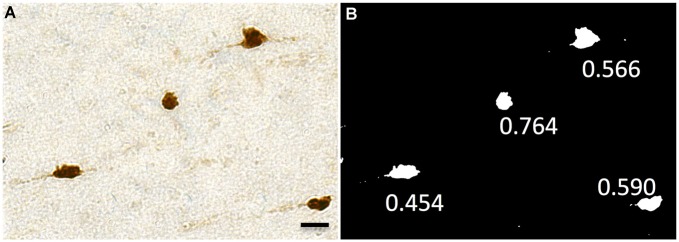
**Representative images showing circularity of neurons.** This ranges from 0 (a flat line) to 1 (a perfect circle). **(A)** Neu-N labeled WMNs. **(B)** These are thresholded and binarized, and circularity is measured with ImageJ. Scale bar = 20 μm.

For soma area and perimeter (Figures 6A–D) the interaction between the factors (age, strata, and ROI) and multiple comparisons with Bonferroni adjustments revealed that soma size in aged animals was significantly smaller in FR and Par, but larger in TE in the superficial zone; this age difference in soma size was not observed in the deep zone. In terms of circularity, somata from older monkeys in FR and Par were more circular in superficial zone but no age differences were observed in TE. In the deep zone, no age difference was observed in circularity (Figures 6E,F, and Table 3). Examples of circularity differences are illustrated in Figure 7.

**Table 3:** **Morphometric analysis of WMNs in non-human primates (NHP) normal aging**.

		Superficial Layer			Deep Layer		
		Area (μm^2^)	Perimeter (μm)	Circularity	Area (μm^2^)	Perimeter (μm)	Circularity
Young	Frontal	342.89 ± 4.40	89.30 ± 1.10	0.735 ± 0.006	136.33 ± 11.60	59.79 ± 2.90	0.778 ± 0.015
	Parietal	310.40 ± 3.90	90.12 ± 0.96	0.768 ± 0.006	123.36 ± 8.86	59.15 ± 2.21	0.818 ± 0.011
	Temporal	197.75 ± 3.42	88.30 ± 0.87	0.792 ± 0.004	132.98 ± 8.14	57.23 ± 2.03	0.821 ± 0.010
Old	Frontal	259.36 ± 4.20*	80.17 ± 1.00*	0.798 ± 0.005^#^	137.68 ± 11.20	63.49 ± 2.80	0.798 ± 0.014
	Parietal	226.51 ± 4.00*	74.83 ± 1.01*	0.799 ± 0.005^#^	131.07 ± 8.50	60.82 ± 2.12	0.818 ± 0.011
	Temporal	263.22 ± 3.47^#^	81.80 ± 0.86^#^	0.789 ± 0.004^#^	165.27 ± 8.01	70.37 ± 1.99	0.793 ± 0.010

Results showed no significant differences in morphological characteristics (area, perimeter, and circularity) of deep WMNs. However, in the superficial zone, WMNs were smaller (area and perimeter) in FC and Par in the older cohort of the monkeys, while in TE, superficial WMNs were larger in the older monkeys (*p < 0.05). In addition, WMNs in the superficial zone in the older monkeys were more circular in FC and Par, but less in TE (^#^p < 0.05).

### Qualitative Observations

**Figure 8:**
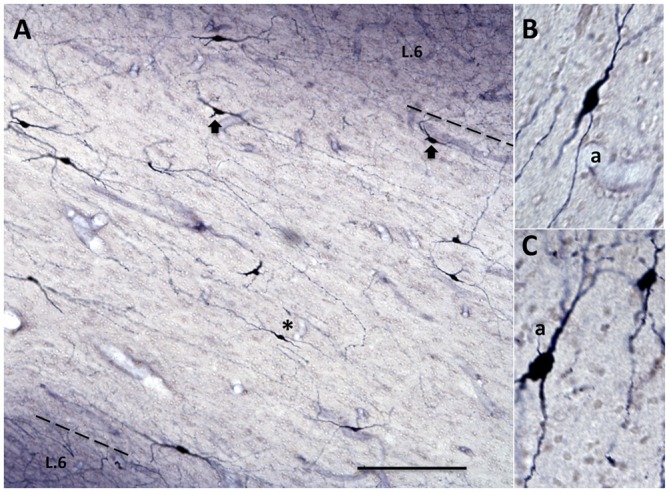
**Subpopulation of inhibitory WMNs, visualized by NADPH-diaphorase. (A)** WMNs in the temporal ROI, between the upper bank of the superior temporal sulcus and the lower bank of the lateral sulcus (medial is at the right). Note extensive dendritic trees and different soma size and shape. Dashed line indicates border of layer 6 (L.6) and white matter. **(B)** Higher magnification of a fusiform neuron (from asterisk in **A**), and in **(C)** of fusiform and granular neurons (from a ventrally adjacent field). a = axon. Scale bars = 200 μm in **(A)**; 50 μm in **(B,C)**.

Heterogeneity of soma size and shape, as reported for the Neu-N material (above), is more clearly evident in the Golgi-like images produced by NADPH diaphorase (Figure 8). These reveal a mix of small granular, fusiform, multipolar, or pyramidal shapes. Soma size varies from a diameter of 5–10 μm for small granular neurons to 25–40 μm in the longest axis of fusiform neurons.

## Discussion

The importance of WMNs, during the subplate stage of early development, is well-established across species in axon guidance and cell migration (Kanold and Luhmann, 2010; Hoerder-Suabedissen and Molnár, 2015); and multiple studies, especially in humans, have further investigated the perinatal and early postnatal reorganization of WMNs in terms of density and subtypes (Delalle et al., 1997; Judaš et al., 2010; Kostović et al., 2014). Other reports, again mainly for human cortex, have documented differential distribution in the adult according to regions (García-Marin et al., 2010), in relation to sulcal depth and gyral crown (Delalle et al., 1997; Kostović et al., 2014), or in cases of neuropathology (Connor et al., 2011; Fung et al., 2011; Kostović et al., 2014).

By contrast, except for several ground-breaking early reports (Kostović and Rakic, 1980, 1990; Smiley et al., 1998), there have been relatively few data for NHP, both in development and adult (e.g., Burke et al., 2009; Fung et al., 2011), even though the NHP brain, importantly, is more accessible to experimental intervention than the human brain. Thus, it is not surprising that so little is known about the function of WMNs in the adult primate. Besides a likely involvement of at least one subpopulation in vascular regulation (see literature in Rockland and Nayyar, 2012 and, in rat, Barbaresi et al., 2014), other functions remain hypothetical, including neurosecretion (Kondo et al., 2015) and neuron-glia or neuron-glia-white matter interactions (Matute, 2011; Fields, 2015). In development, but not necessarily later on, subplate neurons are interconnected by both chemical and electrical synapses, and are believed to be important for synchronous oscillatory network activity (Kanold and Luhmann, 2010).

The present investigation of WMNs in the NHP, as a first step in further investigations, set out to evaluate and compare the density of superficial and deep WMNs in several association areas of four young adult and four aged rhesus monkeys. One main result is that neither the superficial nor deep WMN population show significant neuronal loss in aging, although a moderate decrease was noted for deep WMNs in the frontal cortical zone of the old monkeys. This result is consistent with other studies, where no cell loss could be found with normal aging. In addition to multiple studies of cortical gray matter (for NHP: Hof et al., 2000; Giannaris and Rosene, 2012; and for human temporal cortex: Barger et al., 2015), several studies of human WMNs reported no cell loss in aged subjects. García-Marin et al. (2010), using Neu-N IHC comparable to the present investigation, reported no correlation between age and density of WMNs in their survey in postmortem human white matter beneath areas 10, 17/18, 20, and 24; but only three of their 23 specimens were over 45 years old. Ang and Shul (1995) carried out an analysis of a peptidergic subpopulation in the white matter below striate cortex in the postmortem brains of eight Alzheimer’s patients and seven controls ranging from 56–84 years old, and found evidence of cell loss only in the AD brains. One study, Bu et al. (2003), did report subpopulation-specific cell loss of NADPH-d positive and AChE-positive WMNs “in some cortical areas” (*n* = 11), with nine brains from individuals older than 45 years. As far as we know, there have been no studies of WMN cell loss in aged rhesus monkey or other NHPs, although further analysis, with a larger sample size and smaller ROIs would be desirable.

A second result of the current study is from morphometric analyses of soma size, perimeter, and circularity which showed both area and age effects. For circularity, neurons in FC and Par showed greater circularity in aged monkeys, but those in TE were more circular in young adults. For soma size, superficial WMNs neurons in FC and Par were larger in young adult monkeys. In contrast, Barger et al. (2015), reported that pyramidal neuron soma and nuclear volume are reduced with age in human temporal areas. This could be a species difference, a gray- white-matter difference in the age-response or difference in the regions sampled. Age-related changes in gray matter neurons have been commonly found; for example, in dendritic branching complexity, total spine density, synaptic distribution, and reduction in microcolumnar regularity in area 46 (Cruz et al., 2004; Luebke et al., 2015; among others). Age-related changes in similar parameters remain to be investigated for WMNs.

### Comparison with Other Quantitative Studies

Quantification of WMNs has been undertaken in normal (García-Marin et al., 2010) and schizophrenic (Joshi et al., 2012; reviewed in Connor et al., 2011) postmortem human tissues; and WMN density has been investigated in a developmental series in humans and macaque monkeys (Fung et al., 2011).

Quantitative results can be expected to vary across studies due to differences in sampling protocols and variability in the designated ROIs, but in fact are overall in range. Thus, García-Marin et al. (2010), specify that superficial WMNs are four times as numerous as those in the deep white matter (2169 neurons/mm^3^ vs. 525 neurons/mm^3^). Using a counting box of 0.47 × 0.47 mm, Eastwood and Harrison (2003) report for temporal cortex of human controls an average per box number of 28 superficial WMNs and 10 deep WMNs (their Table 2). One study in the frontal lobe of vervet monkey reports 255 ± 14.4 neurons/mm^2^ (Burke et al., 2009).

### Regional Diversity

A third result of this study is the demonstration of regional diversity. In our sample, the distribution of WMNs was similar in temporal and parietal zones, but lower in the frontal zone. Other investigators have reported regional variability. An early study by Smiley et al. (1998), demonstrated that WMNs, as identified by immunoreactivity for M2-muscarinic receptors, were evenly distributed throughout subdivisions of macaque monkey cortex except for being more scant in primary visual cortex. More recently, García-Marin et al. (2010), found there to be a lower density of WMNs in the temporal region of human cortex, as compared with frontal or cingulate cortex; and Meyer et al. (1992) also report a higher density of neurons in areas 11 and 4 in human.

Factors that could contribute to regional diversity of cell number, cell types, or age-related vulnerabilities of WMNs include differences in developmental origin (Ma et al., 2013), in afferent or efferent connectivity, and in surrounding white matter environment.

### Qualitative Observations

Beyond the general descriptors of “superficial” vs. “deep” or sulcal and gyral locations, criteria are lacking for finer subdivisions of cortical white matter. Within this general reference frame, WMN soma appear not to have any regular distribution pattern, although distinctive dendritic patterns are readily apparent, especially in the Golgi-like NADPH-d preparations (Barbaresi et al., 2014). In our material, we noted some regularity in soma distribution, where two or three neurons were in close apposition (touching or <10 μm edge-to-edge). Possibly similar clusters of neurons were observed in a postmortem study of WMNs from patients with focal epilepsy, but were thought to be pathological malformations (Loup et al., 2009).

At a different scale, the superficial white matter in NHP, in contrast to the typically distinct, narrow subplate in rodents (Kanold and Luhmann, 2010) was often scalloped and broken, consisting of small interspersed neuron-free and neuron-dense patches.

## Conclusion

We undertook this study with the question of whether there is age-related cell loss of WMNs, possibly resulting from their close association with the vulnerable white matter environment. The present results provide no evidence of cell loss in WMNs, but do demonstrate age-related changes in morphometrics of these neurons. Other age-related morphological, connectivity, or neurochemical changes in WMNs remain to be investigated. If they occur, such changes could have multiple impacts (Suárez-Solá et al., 2009), given the broad range of putative WMN functions in sleep (Kilduff et al., 2011), vascular regulation (Cauli and Hamel, 2010; Barbaresi et al., 2014), neuropsychiatric disease (Connor et al., 2011; Joshi et al., 2012) and both corticothalamic (Giguere and Goldman-Rakic, 1988) and corticocortical circuitry (Tomioka and Rockland, 2007; Friedlander and Torres-Reveron, 2009; Kanold and Luhmann, 2010).

## Author Contributions

FM and KSR wrote the manuscript, designed and conducted immunohistochemical experiments, analyzed the data and prepared illustrations. XW performed the cell counts and digitized the sections. DLR guided overall experimental design and participated in writing the manuscript.

## Conflict of Interest Statement

The authors declare that the research was conducted in the absence of any commercial or financial relationships that could be construed as a potential conflict of interest.
